# Health of the City

**Published:** 1847-08

**Authors:** 


### THE WESTERN JOURNAL.

NEW SERIES.—VOL. VIII.—NO. II.

LOUISVILLE, AUGUST 1, 1847.

### HEALTH OF THE CITY.

For some time past quite an unusual tendency to bowel complaints has been manifest among the adult part of our population. In a majority of cases the disease has been simple diarrhea, but in not a few it has been complicated with dysenteric symptoms, and in one which came under our observation, it assumed the character of congestive fever. We saw the patient, a little girl, as she was recovering from the second chill, and have never met with a case of this fever more distinctly marked.

Our experience in this endemic has been most emphatically in favor of *ice*, which has been allowed to our patients in every instance when it was desired. We have lately noticed in the journals a recommendation of ice in the treatment of dysentery. In the Transylvania Journal, (vol. ix, p. 240,) we reported some cases of that disease, in which ice and effervescing draughts produced the happiest effects, and, ever since, our practice has been to gratify the cravings of our patients for ice and iced drinks, in all forms of gastric and intestinal disease. During the excessively hot weather of the present season we have repeatedly witnessed the most pleasing results from this practice both in children and adults. Called to a patient troubled with sick stomach, before giving any medicine, our invariable rule has been to prescribe ice, until the gastric symptoms were relieved, and not a case has yet come under our notice in which it failed to afford marked and almost immediate relief. Thus premised, a few doses of calomel have been found sufficient in most instances to relieve the complaint. Quinine, of course, is prescribed when the case exhibits a periodic character.

The weather has been dry for three months, and for several days about the middle of July, the thermometer rose as high as 92 deg. in the shade. Fever has not prevailed up to this time. A year ago the case was very different. After an unusually wet season, chills were universal in the suburbs of the city, and these continued to recur occasionally until a late period in the spring. No chills are now heard of in quarters where they were so prevalent last summer.

